# Postero-posterolateral approach in total hip arthroplasty

**DOI:** 10.1007/s00264-020-04679-7

**Published:** 2020-07-17

**Authors:** Mokrane Ait Mokhtar

**Affiliations:** Department of Orthopaedics, Grand Hôpital de l’Est Francilien (GHEF), Jossigny, France

**Keywords:** Posterior, Dual mobility, Dislocation, Total hip arthroplasty

## Abstract

**Introduction:**

Evolving surgical techniques in total hip arthroplasty (THA) have sought to make the surgical procedures safer. This requires having highly reproducible incision landmarks and simplifying the procedures. The postero-posterolateral approach, a very posterior incision in the hip, meets those requirements. However, this has not helped to reduce the post-operative dislocation rate. The aim of this study was to assess the relevance of combining the postero-posterolateral approach and next-generation dual mobility cups (DMC) in terms of dislocation risk.

**Materials and methods:**

One hundred and fifty-eight THA were performed consecutively using the postero-posterolateral approach on 150 patients, by a single surgeon, over a 49-month period (November 2010 to December 2014). All acetabular implants were impacted.

**Results:**

Average length of the incision was 7 cm (6 to 9 cm). Mean duration of the surgical procedure was 75 minutes (40 to 100). Mean blood loss was estimated at 210 cc (25 to 410 cc). All patients could walk with assistance the day before transferring to a rehabilitation centre. There was one posterior dislocation (0.63%), without recurrence.

**Conclusion:**

The straightforwardness and reproducibility of the anatomical landmarks used for the postero-posterolateral approach, added to the stability of the dual mobility cup, result in a safe combination in the therapeutic THA arsenal.

## Introduction

The posterior approach in total hip arthroplasty (THA) offers good exposure and favourable working conditions. However, post-operative dislocation is liable to compromise the outcomes of those arthroplasties [1–4].

Several technical tricks [5–9] have been described to help reduce the dislocation rate, with mixed results. However, the solution may be found in the innovative dual mobility concept.

This is a true technical novelty when it comes to THA options. Indeed, according to several authors, it significantly reduces the post-operative dislocation rate, especially when using the posterior approach [10–16]. This implant’s stability comes from its unique design. The implant consists of two articulations, one which is not constrained, between the acetabular cup and the mobile polyethylene liner, while the other is constrained between the femoral head and the mobile polyethylene liner.

The aim of this study was to determine the relevance of combining the postero-posterolateral approach [17, 18] and a next-generation dual mobility implant in terms of dislocation risk. To this end, we performed a retrospective single-centre cohort study of patients who underwent THA with a dual mobility implant.

## Materials and methods

### Patients

One hundred and fifty-eight THA were performed consecutively using a postero-posterolateral on 150 patients, by a single surgeon, over a 49-month period (November 2010 to December 2014).

Indications for THA were intracapsular fracture of the proximal femur (99 patients including two bilateral cases, 16 Garden III, and 83 Garden IV), primary hip osteoarthritis (47 patients including 4 bilateral cases), avascular necrosis (2 patients including 1 bilateral), and rapidly destructive hip osteoarthritis (2 patients including 1 bilateral).

The analytical data gathered for each patient included age, sex, weight, height, BMI (weight/height^2^), the average American Society of Anesthesiologists (ASA) score, the visual analog scale (VAS) pain score at the third day postoperative, as well as intra-operative and post-operative complications.

### Implants

All the acetabular cups were impacted (Quattro® DMC, Groupe Lépine). The femoral stem (Pavi® stem, Groupe Lépine) was impacted in 153 cases and cemented in five cases. The radiological analysis focused on femoral (varus/valgus/neutral) and acetabular implant position (abduction angle).

### Surgical technique

The patient was in the lateral decubitus position with four supports firmly holding the pelvis (pubic bone anteriorly, buttocks, sternal and dorsal support). Three lower limb positions are used during the postero-posterolateral approach.

**Fig. 1:**
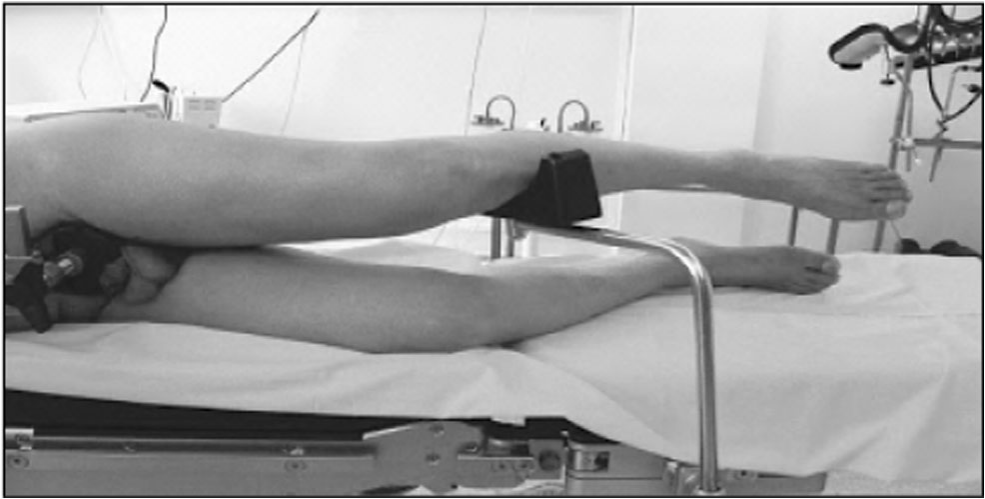
“Lower leg on leg support” position

The extracapsular step was performed with the lower limb in the “lower leg on leg support” position, which required a U-shaped flat support keeping the lower limb in 30° hip flexion and 60° knee flexion (Fig. 1).

**Fig. 2:**
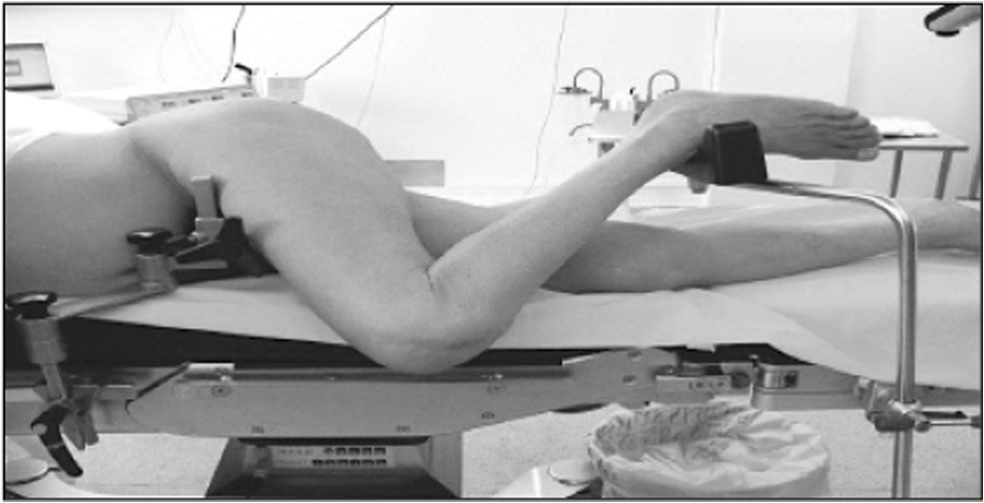
“Ankle on the leg support” position

The acetabular stage was performed in the “ankle on the leg support” position, holding the lower limb in internal rotation and hip adduction with 90° knee flexion. These two lower limb positions do not require operative assistance (Fig. 2).

Finally, for the femoral stage, the lower limb was placed in the conventional position with the knee in 90° flexion and the hip in 90° internal rotation and slight adduction.

**Fig. 3:**
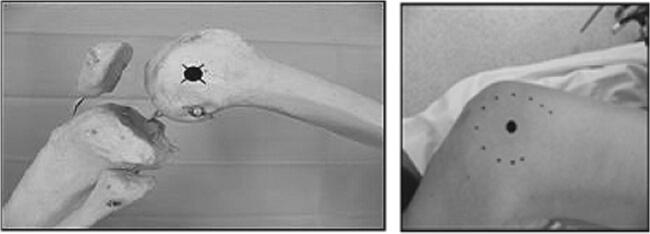
Middle of lateral femoral condyle

**Fig. 4:**
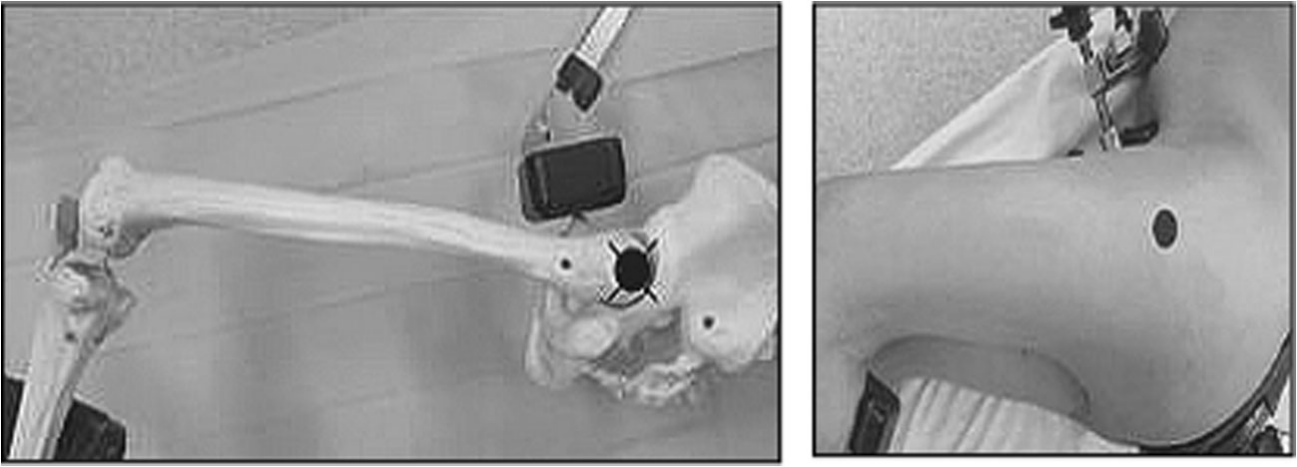
Tip of greater trochanter

**Fig. 5:**
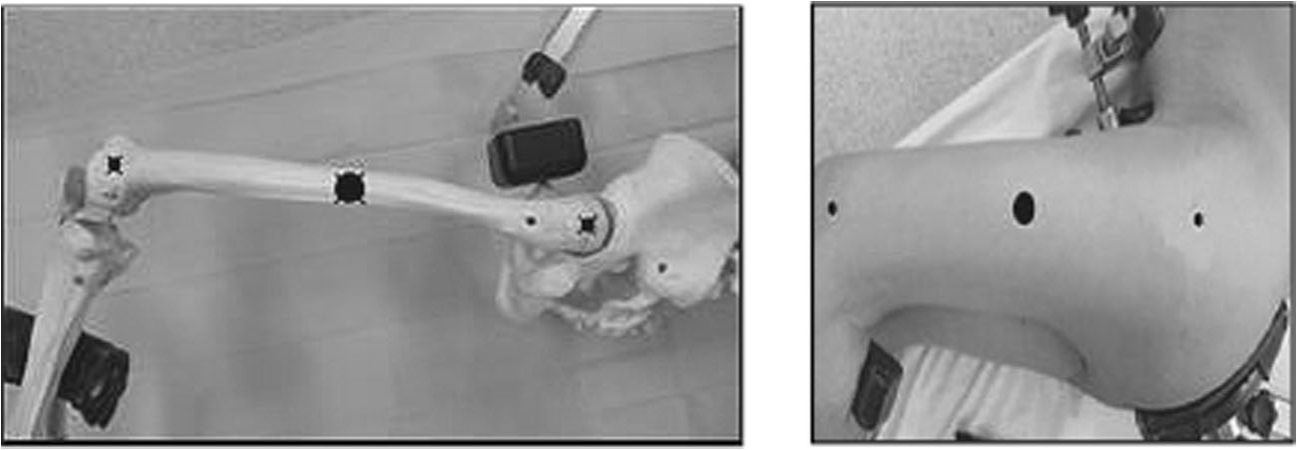
Midpoint between the tip of greater trochanter and middle of lateral femoral condyle

**Fig. 6:**
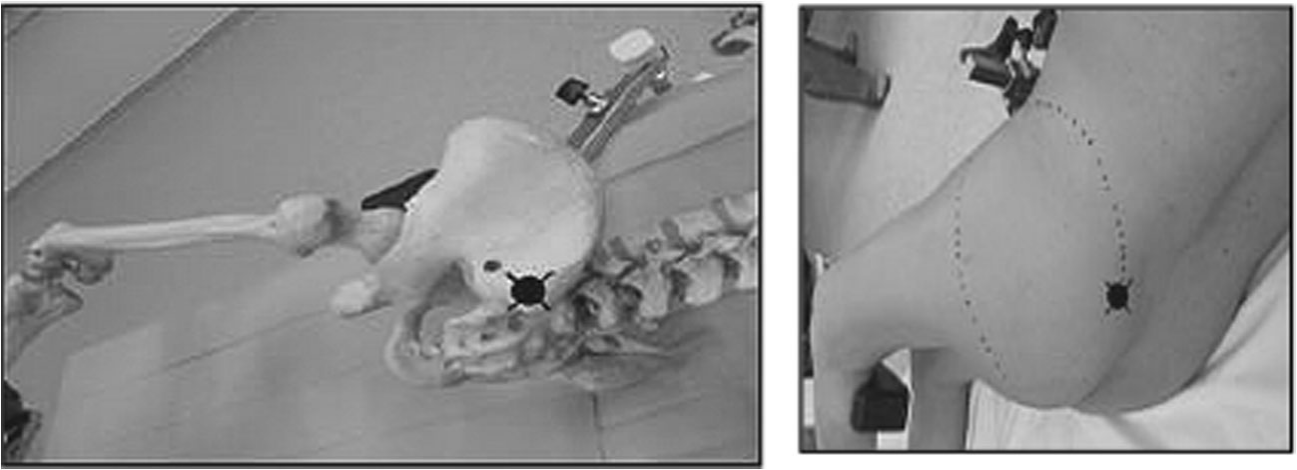
Posterior superior iliac spine

**Fig. 7:**
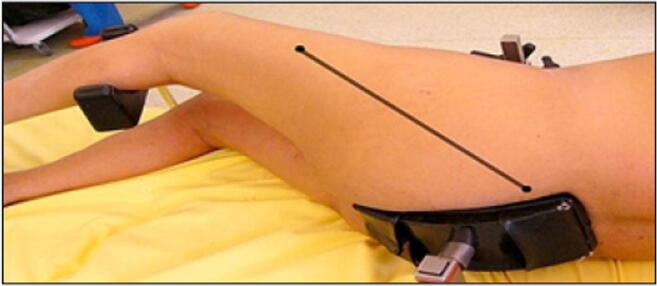
Femoral-iliac line

**Fig. 8:**
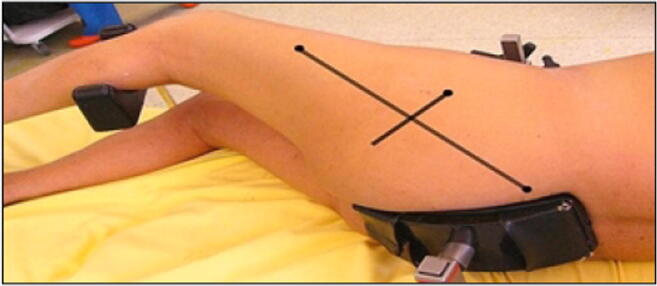
Trochanteric line

**Fig. 9:**
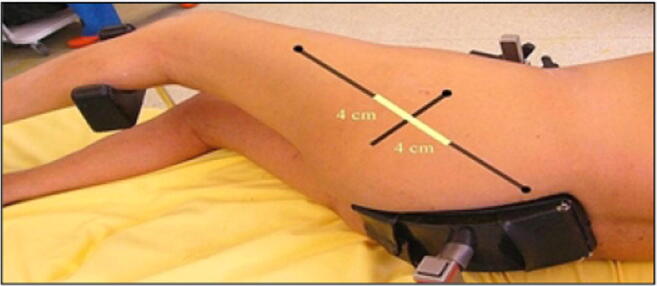
The two orthogonal lines intersect at the proximal and distal starting points of the incision

The incision was traced with the lower limb in the “lower leg on leg support” position, using four bony landmarks and two orthogonal lines. These four bony landmarks were the middle of the lateral femoral condyle (Fig. 3), the tip of the greater trochanter (Fig. 4), the midpoint between the tip of the greater trochanter and the middle of the lateral femoral condyle (Fig. 5), and the posterior superior iliac spine (Fig. 6). These four anatomical landmarks were used to draw two orthogonal lines. The first line was between the posterior superior iliac spine and the middle of the femoral shaft (femoral-iliac line) (Fig. 7), and the second line was perpendicular to the femoral-iliac line going through the tip of the greater trochanter (trochanteric line) (Fig. 8). The skin incision was made on the femoral-iliac line, 3 to 4 cm proximal and 3 to 4 cm distal to the intersection of the trochanteric and femoral-iliac lines (Fig. 9). Optimally, this incision is less than 10 cm (8 ± 2 cm) long.

The extracapsular stage was performed in the “lower leg on the leg support” position.

After making an incision through the subcutaneous tissues, the aponeurosis of the gluteus maximus was open proximally to expose a muscular interstice between the posterior and anterior heads of the gluteus maximus (Fig. 14).

Distally, the superficial muscle fibers of the posterior head of the gluteus maximus inserted on the iliotibial band (ITB) were detached, and then, the ITB was incised distally.

The hip’s external rotators (quadratus femoris, obturator externus, gemellus inferior, obturator internus, gemellus superior muscles) were resected as one block, close to their insertion femoral along with the capsule. The piriformis tendon was kept intact. Finally, the femoral neck was cut with an oscillating saw.

The acetabular part of the surgery was performed in the “ankle on the leg support” position. Conventional reaming of acetabular was done. The trial acetabular cup impacted had to be stable and resist rotational and axial forces.

After reaming the femur with femoral rasps of increasing size, the final trial rasp needed to provide 15 to 20° anteversion and have axial and rotational stability.

With the hip in 90° flexion, the trial implants had to remain congruent with the femur in less than 70° internal rotation.

The definitive femoral and acetabular implants were impacted or cemented. Finally, the hip implant was reduced, and the joint capsule was sutured. No drainage was required.

## Results

The mean follow-up was 38 months (1.5 to 110 months). There were 104 women and 46 men with a mean age of 78 years (range 50 to 98). The BMI was 23.2 kg/m^2^ (14.5 to 28). The ASA score was 2.64 ± 0.60 (range 1 to 4).

The average incision length was 7 cm (6 to 9 cm). The mean operative time was 75 minutes (range 40 to 100). The mean estimated blood loss was 210 ml (40–410 ml). Twenty-nine patients needed a blood transfusion (mean of 2.3 units). The mean VAS pain score at the third day post-operative was 2.15 (0 to 4). There were no intra-operative complications (intra-operative femoral or acetabular fracture, neurovascular trauma).

There was one posterior hip dislocation that appeared during the first week post-operative, after a fall. There were no recurrent dislocation and no other post-operative complications (haematoma, deep infection, deep vein thrombosis).

Assisted walking was possible for all patients the day before leaving for the rehabilitation center or home. The immediate post-operative radiological analysis did not find any more than 3° femoral stem varus. Similarly, the average tilt angle of the acetabular cups, whether cemented or impacted, was 40° (35 to 50).

## Discussion

Minimally hip approaches are defined by a skin incision smaller than 10 cm and sparing the piriformis tendon [19]. In our study, all patients fall under this definition.

Several posterior mini-incision options have been described in the past decades, but two methods stand out. The first is based on a fixed distance, measured in centimeters, starting from the tip of the greater trochanter. The second relies on direct palpation of the trochanter.

In the first method, the fixed distance measured from the tip of the greater trochanter can vary depending on the authors. Swanson’s [20] technique uses 14 cm. Starting 14 cm from the tip of the greater trochanter, in line with the femoral axis, an oblique line is drawn proximally at a 20° angle (this 20° angle is determined by a right angle triangle with an 11 cm hypotenuse and 4 cm height). The incision follows this line and begins 2 cm posterior to the greater trochanter. Other authors propose different measurements: Nakamura [21] recommends 6 to 9 cm, whereas Khan [19] suggests 4 to 6 cm.

**Fig. 10:**
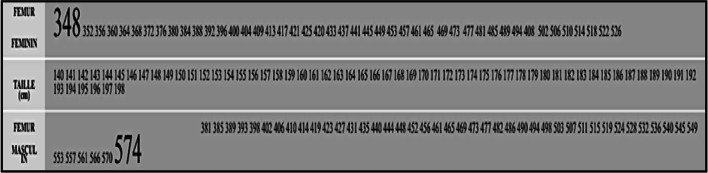
The length of the femur according to the sex and the size of the individual

In an anthropometric study, Olivier [22] determined the length of the femur based on a person’s sex and height. He observed that femoral length can vary up to 20 cm between individuals (Fig. 10).

Therefore, a skin incision starting 10 cm from the tip of the greater trochanter and following the femoral axis will not always provide the same exposure, depending on whether the patient is 181 cm or 152 cm tall. Making an incision based on a predetermined measurement from the tip of the greater trochanter is subject to the variations associated with an individual patient’s femoral length. This affects the reproducibility of these incisions whose precision determines the feasibility of the deep portion of the surgery [23]. The variability in femoral length from one patient to another argues in favour of an incision line that relies on femoral bony landmarks, irrespective of morphotype.

The second method to determine mini-incision positioning depends on precise palpation of the greater trochanter. Many authors rely solely on the protrusion of the greater trochanter to start their skin incision, while others associate it with oblique angles varying between 10 and 45° [24–33]. However, in patients with a large amount of subcutaneous fat at the greater trochanter level, palpation can be difficult or even imprecise. These challenges can make it impossible to reproduce the incisions as described by these authors. Furthermore, they can also lead to inaccurately positioned incisions, affecting not only the quality of exposure but also the surgery itself when it comes to femoral and acetabular preparation.

The incision in the postero-posterolateral approach, whether conventional or minimally invasive [17, 18], does not depend on morphological characteristics, which can vary between individuals, and remains immune from individual specificities involved in palpating the greater trochanter. It uses four easily identifiable bony landmarks to draw two orthogonal lines whose intersection becomes the proximal and distal starting point for the incision. The reproducibility of this incision relies on these four specific bony landmarks, making it possible, regardless of patient morphology, to always achieve the same acetabular and femoral exposure.

The span of an open hand can be used to locate the middle of the femoral shaft. This is a useful landmark which helps counter the variability in femoral size between individuals. The posterior superior iliac spine can easily be identified because it follows the front to back palpation of the iliac crest when the lower limb is positioned with the ankle on the leg support. Furthermore, the line between the posterior superior iliac spine and the anterior superior iliac spine forms a 15° angle with a line perpendicular to the longitudinal axis of the pelvis. This can help locate the posterior superior iliac spine if needed.

The postero-posterolateral approach is markedly offset posteriorly compared with the standard posterolateral approach described by Moore [34]. This more posterior topography gives the postero-posterolateral approach several unique features.

**Fig. 11:**
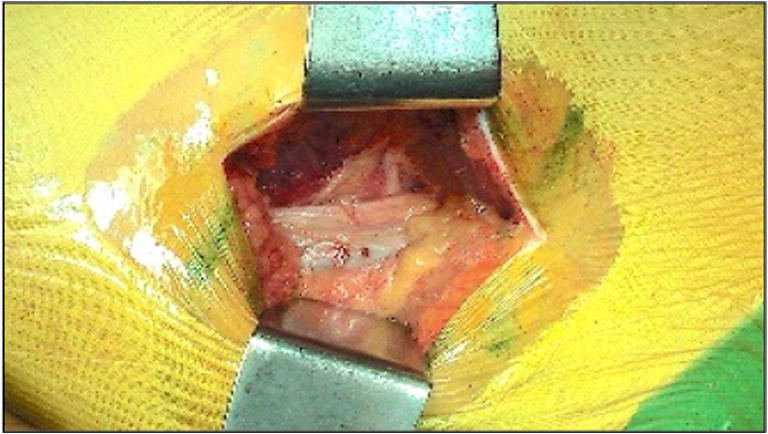
View of the sciatic nerve (extracapsular exposure step)

First is the exposure of the sciatic nerve. During the extracapsular exposure phase, with the “lower leg on the leg support” position, the sciatic nerve is properly visualized (Fig. 11), making it easy to protect during this step.

**Fig. 12:**
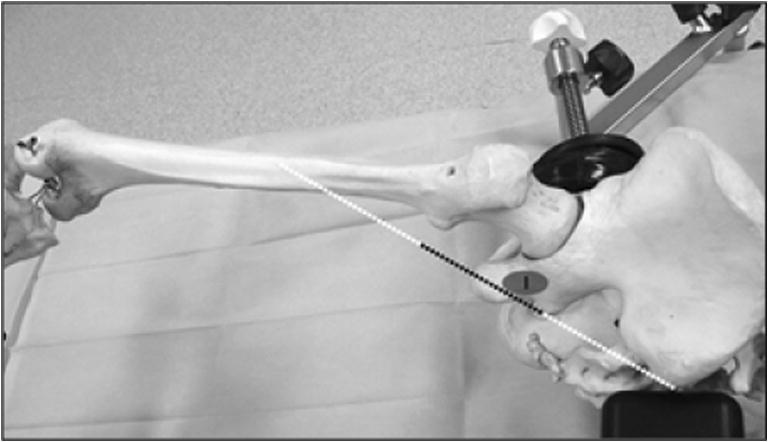
Location of the sciatic nerve in the “lower leg on leg support” position (spot 1)

The projection of the very posterior skin incision crosses the path of the sciatic nerve (Fig. 12), which explains why it is easy to see during the extracapsular exposure phase.

**Fig. 13:**
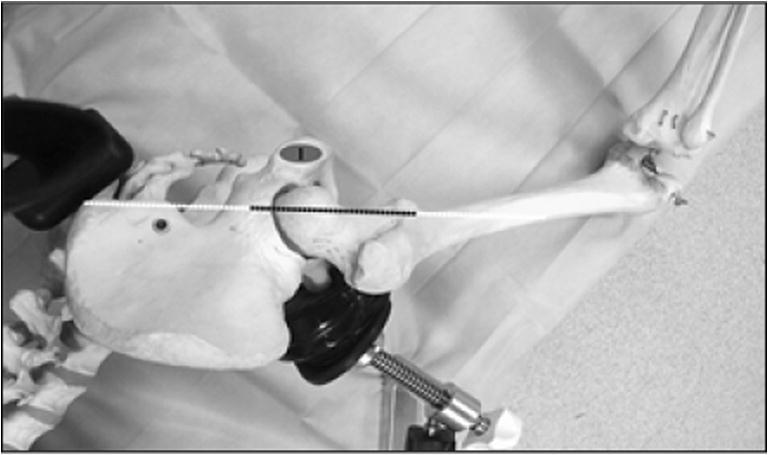
Location of the sciatic nerve in the “lower leg on leg support” position (spot 1)

The sciatic nerve is also protected from any surgical trauma during the femoral and acetabular preparation because the position of the lower limb shields it from any potential damage. While working on the acetabulum with the ankle on the leg support, the sciatic nerve shifts below and posterior to the osseous layer of the lateral aspect of the posterior part of the ischiopubic branch, thus placing the sciatic nerve far from the acetabular working area (Fig. 13).

The same holds true for the femoral preparation, where the sciatic nerve is at a safe distance from the sectioned femoral neck. In our study, there were no complications associated with the sciatic nerve.

**Fig. 14:**
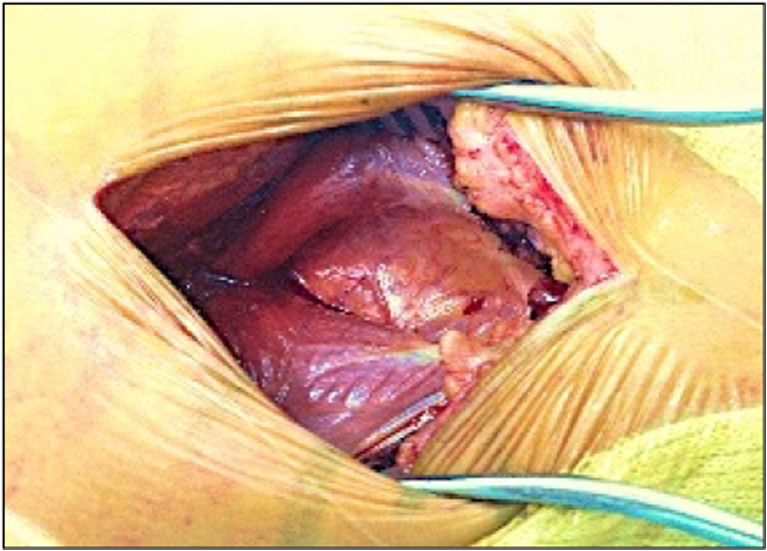
Posterior and anterior heads of the gluteus maximus

**Fig. 15:**
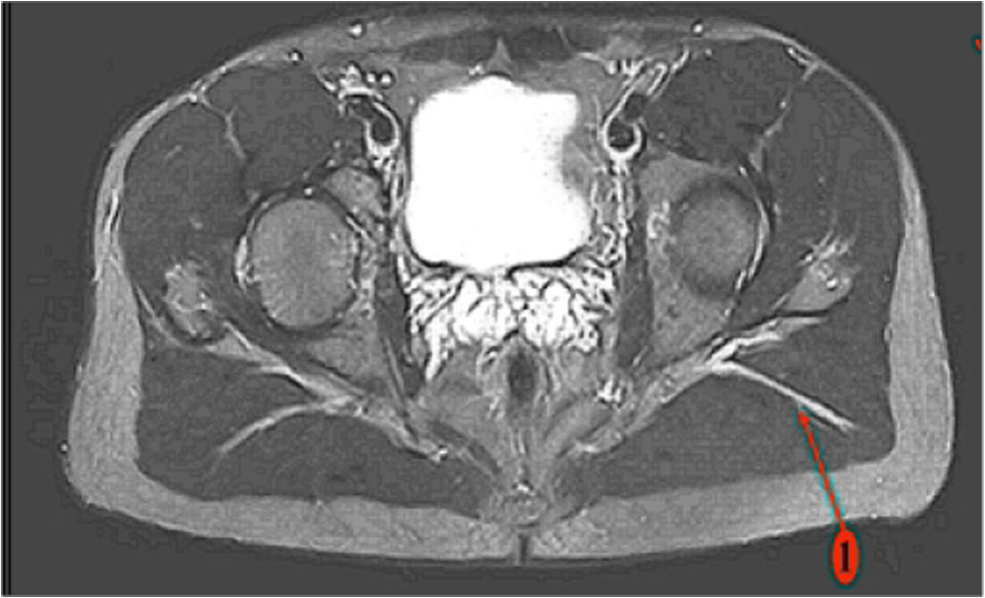
Posterior and anterior heads of the gluteus maximus (spot 1) (MRI image)

The second distinctive feature of the postero-posterolateral approach is anatomical. This very posterior incision provides a passage between the anterior and posterior heads of the gluteus maximus (Figs. 14 and 15). It is essential to stay aligned with the initial skin incision and stay perpendicular to the gluteus maximus muscle, before proceeding proximally to open the aponeurosis of the gluteus maximus.

Passing through this gap confirms that the postero-posterolateral incision is correctly positioned and precedes the hip’s external rotators coming into view.

**Fig. 16:**
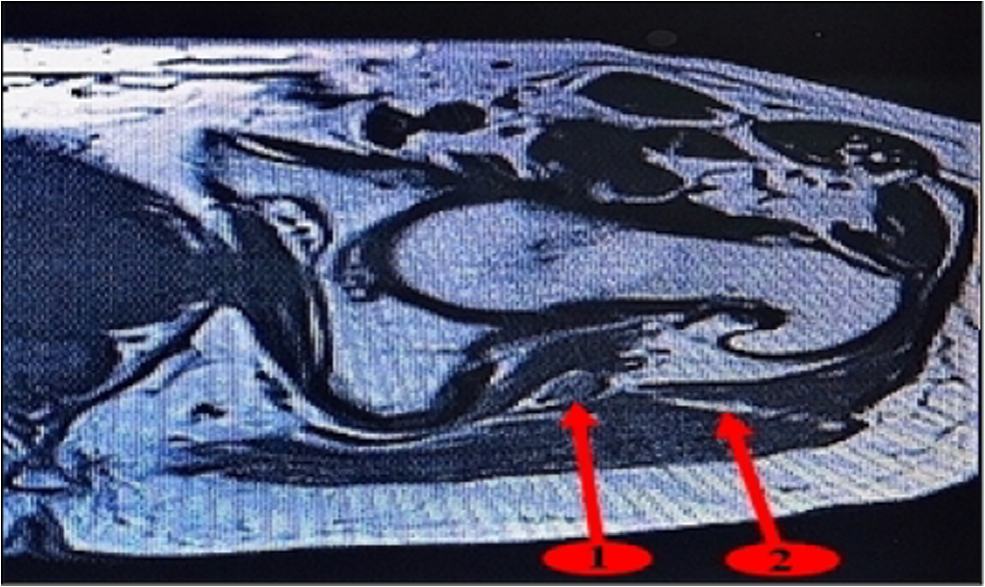
Muscular interstice between the posterior and anterior heads of the gluteus maximus (spot 2) and its proximity to the sciatic nerve (spot 1) (MRI image)

Magnetic resonance imaging (MRI) analysis shows the gap between the posterior and anterior heads of the gluteus maximus. It also confirms the very posterior character of this muscular gap, which can be followed deeply to the sciatic nerve area (Fig. 16).

**Fig. 17:**
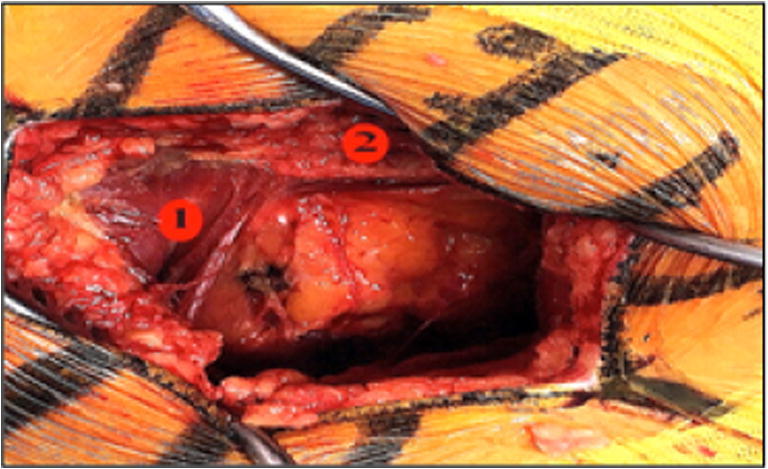
Superficial muscle fibers of the gluteus maximus posterior head (spot1) inserted on the ITB (spot 2)

After opening the aponeurosis of the gluteus maximus proximally and separating the anterior and posterior heads of the gluteus maximus, we find distally where the superficial muscle fibers of the posterior head of the gluteus maximus are inserted on the ITB. These muscular fibers are angled posteriorly and inferiorly to form a roughly 45° angle with the incision axis (Fig. 17). They need to be detached from the ITB before making the distal incision in the ITB.

**Fig. 18:**
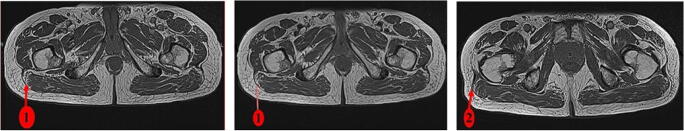
Superficial muscle fibers of the posterior head of the gluteus maximus at different levels of the femoral head-neck complex

MRI images show the superficial muscle fibers of the posterior head of the gluteus maximus opposite to the distal half of the femoral head-neck complex (Fig. 18 spot 1) and its absence beyond the proximal half (Fig. 18 spot 2). Beyond the proximal half of the femoral head-neck complex, the muscular interstice between the posterior and anterior heads of the gluteus maximus is directly under its aponeurosis.

It should be noted that minimally invasive surgical techniques have a long learning curve [23, 24, 27, 35–37]. The straightforwardness of the postero-posterolateral incision considerably reduces this learning curve in everyday practice, thanks to the easily reproducible incision line, regardless of different morphometric characteristics or how well the greater trochanter can be palpated. The minimally postero-posterolateral incision can be used on any patient, without prior eligibility screening [23, 38, 39].

These incision landmarks can be used for a minimally hip approach but, if needed, can also be used within the framework of a traditional open postero-posterolateral incision, extending over 10 cm, with the same exposure benefits.

The postero-posterolateral incision may need to be extended when there is a large quantity of subcutaneous fat, which could cause tissue damage when using the femoral or even acetabular instrumentation. Although the quality of exposure is not affected by the smaller incision, the intraoperative work, especially on the femoral side, can require an extended incision to avoid damaging the skin tissue as the femoral rasps are inserted at the proximal end of the incision.

**Fig. 19:**
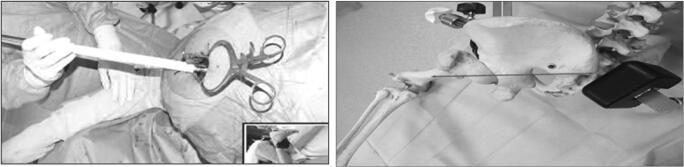
The axis of skin incision and the working axis of the acetabular instrumentation are the same

**Fig. 20:**
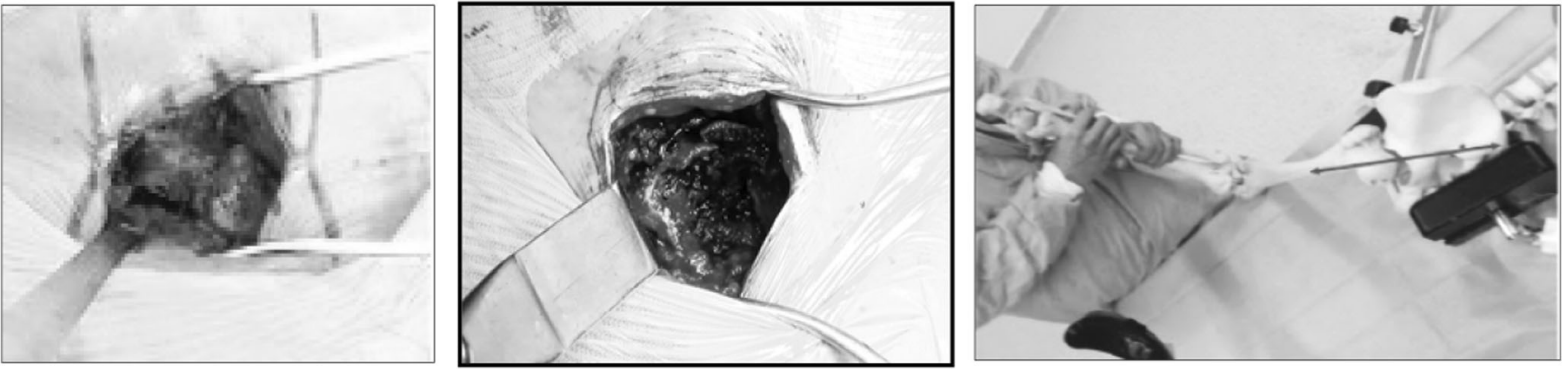
Femoral exposure of the surgical site

The postero-posterolateral approach decreases the risk of being off-course, femoral fracture, or skin trauma, since the cutaneous incision axis and the working axis for the femoral and acetabular instrumentation are the same. The incision line is precisely aligned with the acetabulum when the lower limb is placed with the ankle in the leg support, providing optimal view and access to the acetabulum (Fig. 19). Indeed, the incision axis and the femoral canal working axis are the same (Fig. 20).

Nonetheless, the postero-posterolateral technique is a posterior approach to the hip; thus, postoperative dislocation of the implants remains a concern in everyday practice. Dual mobility cups seem to significantly lower the dislocation risk compared with conventional acetabular cups. Several recent studies have underscored the advantages of using this type of implant to prevent dislocation in revision surgery for recurrent instability [12, 15, 40–46] but also in primary THA [10–16, 40, 42, 47–55], with dislocation rates of 1.5 to 3.8% and 0 to 2.7% respectively.

In our study, there was one postoperative dislocation (incidence of 0.63%), which is consistent with recently published data.

Therefore, using this postero-posterolateral approach in combination with a dual mobility acetabular cup provides this posterior approach with the combined benefits of implant stability and of straightforward, reproducible anatomical landmarks.

## Conclusion

The postero-posterolateral approach provides adequate exposure and view of the surgical site, as well as a comfortable acetabular and femoral working space to ensure the implant is inserted safely and optimally.

The precision of the skin incision is essential to making the deeper surgical steps feasible and reproducible. Indeed, an inaccurate initial incision will not only affect the quality of exposure but also the working conditions for the acetabular and femoral preparation.

The postero-posterolateral approach uses reliable and reproducible bony femoral and acetabular landmarks. This gets around the discrepancies associated with different morphotypes or the need for precise trochanter palpation. Posterior implant dislocation remains a complication with the posterior approach to the hip. Using a dual mobility cup helps significantly reduce the post-operative dislocation rate. Combining the postero-posterolateral approach and a dual mobility cup constitutes a useful adjunct to the available therapeutic options for THA.
